# State Emergency Department Opioid Guidelines: Current Status

**DOI:** 10.5811/westjem.2016.12.30854

**Published:** 2017-03-07

**Authors:** Robert I. Broida, Tanner Gronowski, Andrew F. Kalnow, Andrew G. Little, Christopher M. Lloyd

**Affiliations:** * US Acute Care Solutions, Risk Management Department, Canton, Ohio; † Doctors Hospital, Department of Emergency Medicine, Columbus, Ohio

## Abstract

**Introduction:**

The purpose of this study was to evaluate and categorize current state-sponsored opioid guidelines for the practice of emergency medicine (EM).

**Methods:**

We conducted a comprehensive search of EM-specific opioid prescribing guidelines and/or policies in each state to determine current state involvement in EM opioid prescribing, as well as to evaluate some of the specifics of each guideline or policy. The search was conducted using an online query and a follow-up email request to each state chapter of ACEP.

**Results:**

We found that 17 states had emergency department-specific guidelines. We further organized the guidelines into four categories: limiting prescriptions for opioids with 67 total recommendations; preventing/diverting abuse with 56 total recommendations; addiction-related guidelines with 29 total recommendations; and a community resources section with 24 total recommendations. Our results showed that current state guidelines focus on providers limiting opioid pain prescriptions and vetting patients for possible abuse/diversion.

**Conclusion:**

This study highlights the 17 states that have addressed opioid prescribing guidelines and categorizes their efforts to date. It is hoped that this study will provide the basis for similar efforts in other states.

## INTRODUCTION

Opioid prescriptions and use are of major concern for both the health profession and the general public. The Centers for Disease Control and Prevention reports that 259 million prescriptions for opioid-pain medications were written in 2012, as well as 16,235 opioid-related deaths in the U.S. in 2013.^1^ Headlines featuring opioids fill both general news outlets and medical literature, painting two stories that seem to be at odds with each other and placing the emergency physician (EP) in a nearly untenable position. The need to recognize and manage pain must be balanced with the knowledge that excess opioid prescriptions are leading to a near epidemic in both medication seeking and abuse.^2^ This epidemic and its effect on communities across America has been receiving increased attention by the public, being highlighted by multiple media outlets and has recently become a significant topic during the current election cycle.^3^

While this issue confronts all medical providers, emergency medicine (EM) practitioners are at the nexus of the growing use of prescription pain medication and the devastating consequences of opioids, with nearly 43% of emergency department (ED) visits being related to pain.^4^ According to one study, there were nearly 750,000 ED visits for opioid overdose alone from 1993–2010, while another reported a 14% increase in the number of opioid prescriptions written in the ED from 1993 – 2005.^5^–^6^

Use of guidelines has been shown to decrease use of opioid pain medication in minor and chronic complaints in acute care settings.^7^ To help guide the difficult balancing act of adequately and compassionately treating pain while minimizing diversion/abuse of opioid prescriptions from the ED, the American College of Emergency Physicians (ACEP) has established both policy statements and clinical policies regarding the treatment of acute pain and prescribing of opioid pain medications. Other societies, such as the American Academy of Emergency Medicine (AAEM), offer guidelines to aid in the responsible prescribing of opioids for EPs. Just as the majority of state guidelines have significant overlap, we found the various society guidelines to be similar. As part of ACEP’s policy statement “Ensuring Emergency Department Patient Access to Adequate and Appropriate Pain Treatment (2012),” ACEP leaves it to the individual state chapters to establish guidelines and/or protocols for the treatment of pain in the ED.^8^ Establishment of these guidelines and protocols can assist EM providers in treating pain in a safe and reasonable manner.

## METHODS

We conducted a directed but simple search of EM-specific opioid-prescribing guidelines and/or policies in each state to determine current state involvement in EM opioid prescribing, as well as to evaluate some of the specifics of each guideline or policy. The search was conducted using an online query and a follow-up email request to each state chapter of ACEP. To perform the online search we used the term “ED opioid guidelines” and “emergency department opioid guidelines” for each state (e.g. “Ohio ED opioid guidelines) and evaluated the links that the search returned using the Google search engine. The District of Columbia, Puerto Rico, and national Government Services were omitted from this search as we focused on state-specific guidelines. To standardize the search we limited analysis to the first four pages of results, noting that 91% of online searchers do not click past the first page of search results.^9^ Within these parameters, we identified any guideline that pertained to the ED, whether produced by the state itself or a society/chapter. In addition, we directly contacted each state ACEP chapter executive director by email requesting this same information and sent a second, follow-up email two weeks later. Of the states that had both online search hits and an email response, we did not find significant conflicting information. Only results that specifically pertained to the ED, EM providers or that addressed the treatment of acute pain were considered relevant to this study. We organized all results into a spreadsheet, grouped by type of guideline.

## RESULTS

We found that 17 states had ED-specific guidelines based on our online search and email inquiry. A total of 20 states responded to the email query; of those, 11 had clear guidelines (AZ, CA, DE, HI, NY, OH, OK, OR, PA, RI, WA), an additional four had policy statements or more vague recommendations (KY, NC, TX, WV), and five had no recommendations (CO, MI, NE, NJ, VA). We further organized these into four categories, sorting each category by the most frequently recommended guidelines. For initial categorization, Washington State’s prescribing guidelines were used as the authors were familiar with this guideline and felt it to be a good representation of a comprehensive guideline at this time. We added additional categories that were common across multiple states. The table is a summary of the overall results, displaying which guidelines were recommended by each state. In the Limiting Prescriptions for Opioids section (67 total recommendations), prescribing short-acting formulations and using only short courses were the most recommended guideline. The Preventing/Diverting Abuse (59 total recommendations) sections had frequent recommendations for avoiding replacement of lost prescriptions, utilization of prescription-drug monitoring programs, and requirements for government-issued ID to receive an opioid prescription. Addiction-related guidelines (34 total recommendations) encouraged screening tools and avoidance of methadone distribution to patients. Finally, the Community Resources section (24 total recommendations) had suggestions for care coordination programs, educational information for patients, and maintaining a list of available clinics in the community.

**Population Health Research Capsule**

What do we already know about this issue?

The opioid epidemic has had devastating effects. As emergency physicians we are on the front line, forced to address acute and chronic pain in a conscientious manner.

What was the research question?

Are state guidelines available to help facilitate emergency physicians’ appropriate use of opioids?

What was the major finding of the study?

Only a few states offer opioid robust guidelines that aid the care of patients in the emergency department.

How does this improve population health?

With robust established guidelines, emergency physicians will be better equipped to use and prescribe opioids appropriately and efficiently.

### Limiting Prescribing of Opioids

This category provided EPs with advice on limiting the number, strength and duration of action of opioids being prescribed. All states combined had 67 different guidelines referring to limiting the prescribing of opioids.

### Preventing/Diverting Abuse

The second category included state attempts to limit diversion or abuse of opioids by ED patients. All states combined had 59 different guidelines referring to preventing and diverting abuse of opioids.

### Addiction-Related

The third category includes various items related to known, suspected or occult misuse or addiction, including both abuse screening and management for known substance abusers. There were 34 guidelines in this category across all of the states.

### Community Resources

Lastly, ED integration with existing community resources was addressed by all the states with 24 guidelines combined between them. Washington State has been a leader in establishing state guidelines on opiates, and had the most guidelines of any state, numbering 16.^10^ Arizona and Oregon were next, with 14 each (Table). Arkansas and Ohio rounded out the top five states, with 12 each.

## DISCUSSION

The number of prescribed opioids and deaths related to their use has moved to the forefront of mainstream media, and found their way into both state and federal political discussions. It is important to note that while ED opioid prescribing has risen, the bulk of the opioid problem is due to long-acting or extended-release formulations used in treatment of chronic pain.^6^,^11^ These agents are rarely prescribed in the ED, likely because the majority of painful conditions seen in the ED are acute in nature.^12^

ACEP and AAEM, as well as many other organizations, have been aggressive in the formation of policy and recommendations in regard to EP opioid prescribing and have encouraged individual states to do the same. These investigators found that although several organizations have made recommendations, many states have yet to implement any guidelines. The reasons for this are unknown, nor is it known whether local state ACEP chapters helped to contribute to the overall ACEP guidelines. The authors believe that ACEP likely encouraged individual states to create their own guidelines in an effort to help solidify a universal proper approach, give ED practitioners a second resource, and to help continue to increase awareness of the opioid problem.

The idea behind the simple search parameters was that this theoretically should be something easily accessible and discoverable by practitioners who are seeking the resource. The lack of easily identifiable guidelines via online search was concerning. For a topic that is becoming as mainstream as opioid prescribing, the authors felt that if the most generic search could not find the guidelines then they would not be used in clinical practice.

Review of the guidelines showed that most states were able to craft guideline language that, while discouraging prescription of opioids in the ED, maintained EP professional judgment and autonomy to best address the very real need of their patients in pain. However, a minority of states had ED-specific guidelines, and our research demonstrated that only 17 states had created such guidelines. While most states developed guidelines affecting all providers, these authors found they established relatively few guidelines that would impact EM providers in any meaningful way due to their focus on chronic pain therapy. These chronic-pain prescribing guidelines have been shown to reduce opioid prescriptions, and with proper planning and execution, acute pain guidelines may be able to accomplish the same.^13^

Future research should focus on comparing states that have ED-specific opioid guidelines to those states that have broad guidelines or lack guidelines completely and how these differences possibly impact the rising opioid epidemic. We are not aware of any literature on the exact impact of awareness, prescribing method changes or adherence to guidelines in states that have only national guidelines versus those with state-specific guidelines. Further investigating the comparative impact of opioid prescribing patterns between primary care, inpatient care, and emergency care could help further define the need for ED-specific prescribing patterns.

## LIMITATIONS

There are several limitations of this study. First, there could be states with guidelines that were not easily found via online search or were not provided to us upon request to the ACEP chapters. It is possible that our online search parameters were inadequate or not specific enough to discover the state guidelines. Finally, we only looked at state ACEP guidelines and did not include any states that may have opioid prescribing guidelines from other EM organizations (i.e., AAEM).

## CONCLUSION

This study highlights the various ways in which states have approached opioid prescribing guidelines and categorizes their efforts to date. It is hoped that this study will provide a rational basis for similar efforts in other states or on the federal level.

## Figures and Tables

**Table:** The 17 U.S. states with emergency department (ED) opioid prescribing guidelines. (The remaining 33 states did not have ED-specific opioid prescribing guidelines listed online or received from their ACEP chapters at the time this research was conducted.)

	AZ	AR	CA	DE	FL	HI	MD	ME	MN	NY	OH	OK	OR	PA	RI	UT	WA	Total
Limiting Rx for opioids																		
Only prescribe a short course	X	X	X	X		X	X	X	X	X	X	X	X	X	X	X	X	16
Use short-acting formulations	X	X	X	X		X	X			X	X	X	X	X	X	X	X	14
Parenteral is discouraged for acute exacerbation of chronic pain	X	X	X	X		X		X			X	X	X		X		X	11
Start with lowest effective dose	X					X	X			X		X		X	X	X		8
Meperidine is discouraged	X	X		X							X		X				X	6
Address chronic pain with non-opioids			X						X	X		X		X				5
Avoid opioids with benzodiazepines						X			X	X		X						4
Preventing abuse/diversion																		
Lost Rx- do NOT replace		X	X	X		X	X	X		X	X	X	X	X	X		X	13
Use Rx drug monitoring programs	X		X			X	X	X	X		X	X	X	X	X		X	12
ED Rx should require government ID	X	X	X					X			X		X		X		X	8
Patient should not receive Rx from multiple providers				X			X	X					X	X	X		X	7
Dispense only the amount needed until pharmacy or PCP office	X	X										X		X	X		X	6
ED should photograph pain patients without government ID	X	X						X			X						X	5
Utilize ED information exchange programs																	X	1
Addiction related																		
Should not provide methadone for patients in treatment programs	X	X	X	X		X	X				X		X				X	9
Assess for misuse/addiction with a screening tool	X		X				X	X	X	X		X	X				X	9
Perform urine drug screen if suspicion	X							X	X		X							4
ED should receive pain agreements		X				X											X	3
Encourage/Assist patients in seeking detox														X	X			2
Community																		
ED should use a care coordination program	X	X		X		X		X	X								X	7
Provide patient education information						X	X			X		X	X	X				6
Maintain list of clinics	X	X									X		X				X	5
Reference ACEP Clinical Policy on state site					X							X	X		X			4

*Rx,* prescription; *ED,* emergency department; *ID,* identification.

## References

[1] (2014). Prescription Drug Overdose in the United States: Fact Sheet. Centers for Disease Control and Prevention Website.

[2] Szalavitz M (2011). Report: Chronic, Undertreated Pain Affects 116 Million Americans. Time.

[3] Shallow P (2014). Dying for pain relief in the opioid epidemic. CBS News.

[4] Cantrill S, Brown MD, Carlisle RJ (2012). Clinical policy: critical Issues in the prescribing of opioids for adult patients in the emergency department. Ann Emerg Med.

[5] Hasegawa K, Espinola JA, Brown DF (2014). Trends in U.S. emergency department visits for opioid overdose, 1993–2010. Pain Med.

[6] Brunk D (2008). Opioid Prescriptions Climb for Pain-Related ED Visits. ACEP News.

[7] del Portal DA, Healy ME, Satz WA (2016). Impact of an Opioid Prescribing Guideline in the Acute Care Setting. J Emerg Med.

[8] (2012). Ensuring Emergency Department Patient Access to Adequate and Appropriate Pain Treatment.

[9] van Deursen AJAM, van Dijk JAGM (2009). Using the Internet: Skill related problems in users’ online behavior. Interact Comput.

[10] Washington Emergency Department Opioid Prescribing Guidelines.

[11] Miller M, Barber CW, Leatherman S (2015). Prescription opioid duration of action and the risk of unintentional overdose among patients receiving opioid therapy. JAMA Intern Med.

[12] (2010). Outpatient Prescription Opioid Utilization in the U.S., Year 2000–2009.

[13] Franklin GM, Mai J, Turner J (2012). Bending the prescription opioid dosing and mortality curves: Impact of the Washington State opioid dosing guideline. Am J Ind Med.

